# Thyroid Hormone Effect on the Differentiation of Human Induced Pluripotent Stem Cells into Hepatocyte-Like Cells

**DOI:** 10.3390/ph14060544

**Published:** 2021-06-07

**Authors:** Mariia S. Bogacheva, Margarita A. Bystriakova, Yan-Ru Lou

**Affiliations:** 1 Division of Pharmaceutical Biosciences, Faculty of Pharmacy, University of Helsinki, 00014 Helsinki, Finland; mariia.bogacheva@helsinki.fi (M.S.B.); margarita.bystriakova@gmail.com (M.A.B.); 2 Department of Clinical Pharmacy and Drug Administration, School of Pharmacy, Fudan University, Shanghai 201203, China

**Keywords:** thyroid hormone, hepatic development, human induced pluripotent stem cells, hepatocyte-like cells, liver maturation

## Abstract

Human induced pluripotent stem cells (hiPSCs) hold great potential as an unlimited source for obtaining hepatocyte-like cells (HLCs) for drug research. However, current applications of HLCs have been severely limited by the inability to produce mature hepatocytes from hiPSCs in vitro. Thyroid hormones are one of the hormones that surge during the perinatal period when liver maturation takes place. Here we assessed the influence of thyroid hormone on hepatic progenitor differentiation to HLCs. We analyzed gene and protein expression of early and late hepatic markers and demonstrated the selective activity of thyroid hormone on different genes. Particularly, we demonstrated thyroid hormone-dependent inhibition of the fetal hepatic marker AFP. Our study sheds light on the role of thyroid hormone during liver differentiation and maturation.

## 1. Introduction

Human pluripotent stem cell (hPSC)-derived hepatocyte-like cells (HLCs) represent a promising cell model for drug research [1,2] and various biomedical applications [3,4]. Induction of HLCs in vitro includes the use of soluble factor-based differentiation cocktail and the culture condition which are aiming to mimic the natural liver development in the embryogenesis. It is a multistage process which, first, requires the formation of definitive endoderm (DE), hepatic progenitors, fetal hepatocytes, and finally, HLCs. Every subsequent stage is regulated by the carefully adjusted combinations of growth factors specific for this particular stage of the development [5]. In vivo DE development is initiated by the Nodal signaling [6], which is mimicked in vitro by administration of activin A alone [7], or in combination with the glycoprotein Wnt-3A or bone morphogenetic proteins (BMP) [8,9], or by small molecule sodium butyrate [10], or by the administration of the GSK3 inhibitor CHIR99021 [11].

Furthermore, DE cells undergo hepatic specification using different combinations of BMPs and fibroblast growth factor 4 (FGF4) [5,12,13,14]. The obtained hepatic progenitors are further differentiated using hepatocyte growth factor (HGF) [12]. Additionally, hepatic maturation requires the presence of interleukin-6 family growth factor OSM and synthetic glucocorticoid dexamethasone (DEX) [15]. BMP7 promotes the key liver protein albumin expression [16]. Undesired formation of cholangiocytes can be prevented by inhibiting the NOTCH and Wnt signaling [17].

Differentiation of hPSCs into HLCs in two-dimensional culture includes switching of the culture matrix after the formation of DE cells (usually Matrigel [18,19]) to the matrix which better mimics the extracellular matrix (ECM) of hepatic progenitors [5,13,20]. The existing protocols for in vitro hepatic differentiation of hPSCs often result in an immature cell population, although this research area is actively developing, and many research groups have come up with various improvements [12,21,22] and have assessed different components for the differentiation medium [11].

At birth and shortly after birth, most hepatic functions rapidly elevate due to dramatic changes from intrauterine to extrauterine [20]. Fetus–newborn transition involves endocrine, metabolic, cardiovascular, and lung adaptions [21]. Cortisol, catecholamines, and thyroid hormones (THs) are the main modulators in endocrine adaption [21]. It is well known that THs participate in organ development [22]. The role of THs, including 3,5,3′-triiodothyronine (T_3_) and 3,5,3′,5′-tetraiodothyronine (T_4_), in tissue development and maturation has been demonstrated in detail in bone, brain, intestine, heart, and pancreas. T_3_ was found to induce the maturation of mouse embryonic stem cell (mESC) and human induced PSC (hiPSC)-derived cardiomyocytes [23,24] and promote pancreatic β-cell maturation in rats [25]. It was used to improve the in vitro maturation of hPSC-derived pancreatic β-cells and fetal islet cells [26,27,28]. T_3_ has been shown to induce the differentiation of rat liver progenitor oval cells into hepatocytes [29]. THs are known to be actively involved in the process of liver regeneration [30]. They activate and suppress in summary 55 hepatic genes through the interaction with the nuclear TH receptors (TRs) TRα and TRβ [30]. Thus, THs regulate the metabolism of carbohydrates and fatty acids, insulin action, cell proliferation, immune functions, synthesis of glycoproteins, et al. [31]. In mice, it was shown that T_3_ stimulates the mitotic activity of the transplanted hepatocytes [32] through the TRβ [33].

In early embryo development, the fetus receives THs from the maternal placenta and synthesizes them by itself in the late stages [34,35]. The human placenta is rich in TH transporters, which explains its permeability for THs. THs, in their turn, are actively involved in the processes of tissue differentiation and maturation [36].

The conversion of the inactive T_4_ form of TH into an active one T_3_ is mediated by the tissue-specific deiodinases DIO1, DIO2, and DIO3. DIO3 is a fetal liver enzyme that plays an important role in the protection of the embryo from the excessive concentrations of maternal THs supplied through the placenta [36]. DIO3 activity decreases from fetal to mature liver, being high in preterm infants and absent in full-term infants [35].

We hypothesized that the TH administration to the hPSCs at certain stages of hepatic differentiation can improve the maturity of resulted HLCs. In this study, we conducted the differentiation of hiPSC into HLCs and demonstrated how T_3_ affected the expression of certain markers during the differentiation.

## 2. Results and Discussion

**Figure 1:**
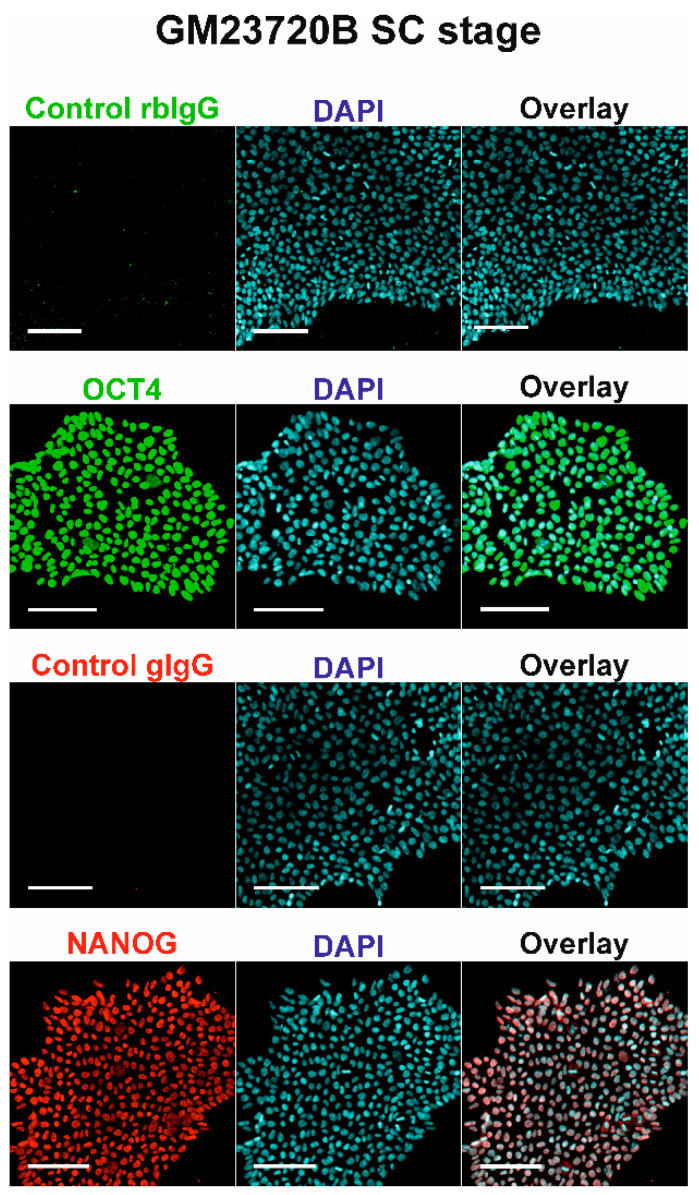
Expression of OCT4 and NANOG proteins in undifferentiated GM23720B cells. Nuclei of cells were stained with DAPI (blue). Proteins of interest were stained either with Alexa Fluor 488 (OCT4), shown in green, or with Alexa Fluor 594 (NANOG), shown in red. Scale bars = 100 μm.

We conducted the hepatic differentiation of hiPSC line GM23720B to study the influence of T_3_ hormone on hepatocyte differentiation in vitro (Figure S1A). Before starting the differentiation, we ensured that GM23720B cells displayed the characteristic stem cell colony organization (Figure S1B) and positive protein expression of the stemness markers OCT4 and NANOG (Figure 1). We implemented the differentiation of the hiPSC into HLCs with and without the treatment with T_3_ hormone at the defined stages of differentiation.

### 2.1. DE Induction

**Figure 2:**
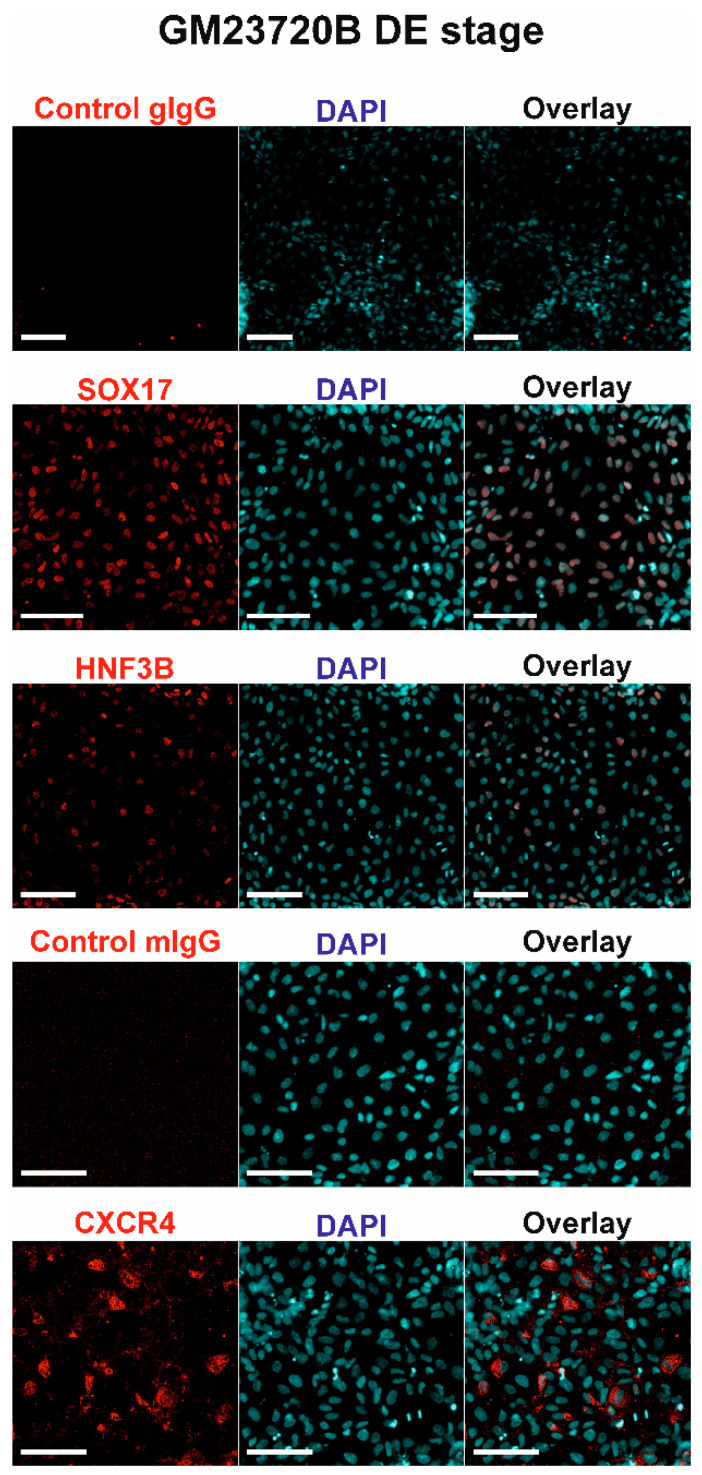
Expression of SOX17, HNF3B, and CXCR4 proteins in GM23720B-derived cells at day 6 (DE stage) of the differentiation. Nuclei of cells were stained with DAPI (blue). Proteins of interest were stained with Alexa Fluor 594 (red). Scale bars = 100 μm.

The first step of hepatic differentiation is the formation of DE cells. Earlier, we have developed a protocol for the hPSC differentiation into DE cells [19]. It involved treatment with AA during six days. This protocol enables obtaining highly viable cells with high expression of DE cell-specific markers and potency to be differentiated into HLCs, and, therefore, has been implemented in the current study. At day 6 of the differentiation, GM-23720B-derived cells acquired characteristic DE morphology and, based on confluency, possessed high viability, allowing further differentiation procedure (Figure S1B, day 6). At day 6 of the differentiation, GM23720B cells became positive with DE cell markers SOX17, HNF3B, and CXCR4 proteins (Figure 2), indicating the efficient DE cell formation.

### 2.2. Hepatic Specification (Generation of Hepatic Progenitors)

The ECM is a dynamic structure and, during embryo development, it remodels itself according to current tissue requirements. Therefore, it is essential to adjust the culture’s physical conditions to mimic the natural development process. It is shown that the hepatic progenitor-like environment is beneficial for the differentiation of DE cells to hepatic lineage [5]. Therefore, at the second step of the differentiation, we transferred DE cells from Matrigel to the LN521 matrix, which was shown to be a suitable matrix for the hepatic specification in a previous study, and followed the previously described procedure for the formation of hepatic progenitors [5]. BMP2, BMP4, and FGF proteins act in a concerted manner, regulating the hepatic specification of DE cells [37]. It has been shown to be effective in in vitro experiments for hepatic induction [5,38]. We modified the culture medium with the growth factor combination of BMP2, BMP4, and FGF4 to mimic the hepatogenesis process. By day 10, we observed the change of morphology: the cells became heterogeneous, and cell types were grouped in clusters, some cells started to acquire polygonal shape (Figure S1B, day 10).

### 2.3. Differentiation and Maturation of Hepatic Progenitors

#### 2.3.1. Optimization of the T_3_ Concentration

T_3_ activates the transcription of genes through the interaction with nuclear TRs that serve as transcription factors in a ligand-dependent manner for the T_3_-responsive genes [39]. T_3_ was shown to induce hepatic differentiation in fetal liver [29] and liver regeneration [30]. We thus hypothesized that T_3_ administration during the expansion and maturation of hepatic progenitors may positively affect the expression of specific hepatic genes and proteins.

To find the optimal T_3_ hormone concentration for the improvement of hepatic maturation, we tested four T_3_ concentrations (1 nM, 10 nM, 100 nM, and 1000 nM) and three administration timing intervals (from day 9 to day 22, from day 13 to day 22, and from day 17 to day 22). We performed hepatic differentiation of GM23720B cells and assessed the influence of different T_3_ concentrations and time of its administration by the level of mRNA expression of the key early, midlate, and late hepatic markers.

Alpha-1-fetoprotein (AFP), a marker of the early hepatic development, is supposed to decrease by the end of the differentiation experiment, meaning the natural switch from fetal stage to the adult stage of hepatocytes. The lowest *AFP* mRNA expression by the end of the differentiation was obtained using the administration of 1000 nM T_3_ from day 9 until day 22 (Figure S2A).

The rise of the expression of midlate marker albumin *ALB* indicates further hepatic differentiation. The highest *ALB* induction in the cells was obtained after the treatment with 1 nM and 100 nM T_3_ from day 13 to day 22 (Figure S2B). The highest level of TH responsive *THRSP* (or *SPOT14*), whose expression characterizes adult hepatocytes, was achieved by the treatment with 1000 nM T_3_ from day 9 to day 22 (Figure S2C). The expression of *CK19*, the marker of the bile duct epithelium and hepatic progenitors, should decrease after the hepatic progenitor stage. *CK19* expression in all the conditions decreased after day 9, though the level at the end of differentiation was higher than that in primary human hepatocytes (PHH). The lowest expression was achieved by treating cells with 10 nM T_3_ from day 13 or 17 to day 22 and with 1000 nM T_3_ from day 17 to day 22 (Figure S2D). T_3_ treatment did not affect the liver-specific marker *AAT* induction, which decreased after day 17 in both treated and nontreated cells (Figure S2E).

We did not find a universal hormone concentration that upregulates midlate and all the late markers and, at the same time, downregulates the early markers. Although the treatment with the highest T_3_ concentration slightly decreased *AAT* expression, it dramatically increased *THRSP* and decreased *AFP*, while *ALB* upregulation depended on the timing of the T_3_ treatment. *CK19* downregulation also relied on the later administration of the moderate concentration of T_3_. Thus, we chose to use 1000 nM T_3_ from day 9 to day 13 and 10 nM T_3_ from day 13 onwards. We observed the hepatic mature and immature gene expression pattern change until day 17 (Figure S3). We found no significant decrease of *AFP* by day 17 (Figure S3A). *ALB* gene expression has increased from day 9 to day 17 without the T_3_ treatment, however, no significant difference was found in *ALB* expression dynamics between T_3_-treated and nontreated cells (Figure S3B). No difference was found between T_3_-treated and nontreated cells in *CK19* and *THRSP* expression (Figures S3C and S3E). *CYP3A4* expression increased after day 13 in both treated and nontreated cells (Figure S3D). We suggest that a 17-day differentiation procedure is not enough for the cells to acquire maturity status, and thus subsequently implemented the 22-day differentiation protocol (Figure S1A).

Distinctive hepatocyte morphology is characterized by polarization, polygonal cell shape, and round nucleus [10]. Each cell should be connected with the adjacent cell through tight junction. By day 22 T_3_-treated and nontreated cells increased in size and developed more flat cell areas. Many polygonal cells contained vesicular structures (Figure S1B, day 22).

#### 2.3.2. Gene Expression Profiles of GM23720B-Derived Cells

The maturity of iPSC-derived HLCs is generally assessed via the analysis of the downregulation of immature markers and the upregulation of mature liver markers.

**Figure 3:**
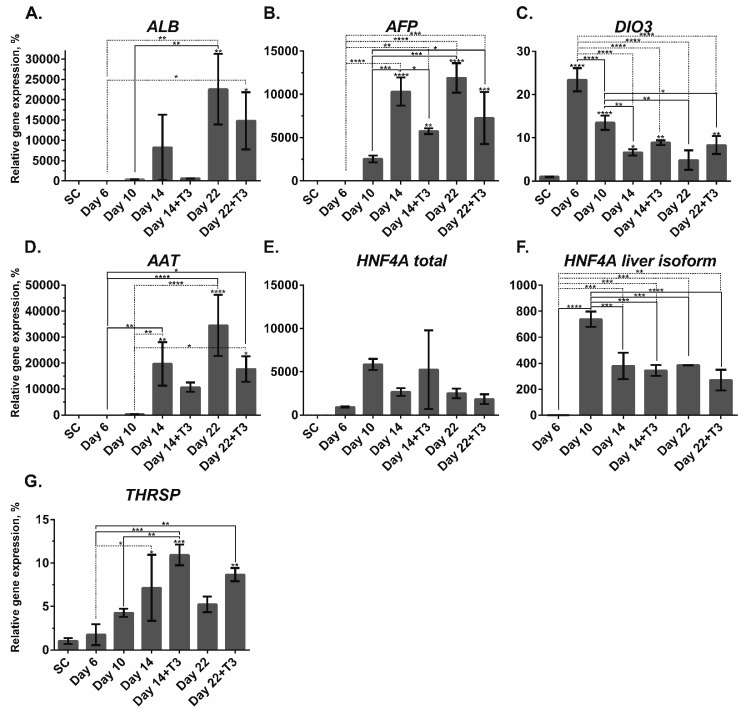
The mRNA expression patterns of the mature hepatic (*ALB*, *AAT*, total *HNF4A*, liver *HNF4A*, and *THRSP*), fetal hepatic (*AFP* and *DIO3*) specific markers during hepatic differentiation of GM23720B cells. Relative gene expression was measured by qPCR and normalized with the *RPLP0* housekeeping gene. Fold inductions were calculated with the reference to the stem cell samples (SC). *n* = 3 biological repeats. Error bars are SD. One-way ANOVA followed by Sidak’s multiple comparisons test was used to compare between any pairs. Statistical significance * adjusted *p* < 0.05, ** adjusted *p* < 0.01, *** adjusted *p* < 0.001, and **** adjusted *p* < 0.0001 in comparison with SC are shown above bars. Statistically significant differences * adjusted *p* < 0.05, ** adjusted *p* < 0.01, *** adjusted *p* < 0.001, and **** adjusted *p* < 0.0001 between days of the differentiation or between the T_3_-containing (+T3) or no T_3_ are shown above lines.

*ALB* mRNA expression significantly increased by day 22 compared to day 10 independently of T_3_ administration (Figure 3A) indicating the absence of T_3_-mediated regulation of *ALB* expression. *AFP* encodes transporter protein AFP. It is known to be expressed by hepatic progenitors, such as oval cells, and fetal hepatocytes. Its expression is inhibited after birth [12], giving away its functions to ALB. An earlier study showed that T_3_ induced rat liver oval cell differentiation into hepatocytes by inducing the expression of HNF4A and reducing the expression of AFP [29]. In our study, *AFP* gene expression level gradually increased after day 10. First, on day 14, we observed a downregulation of *AFP* in cells treated with the T_3_ hormone. By day 22, *AFP* was also downregulated by T_3_, though the difference was not statistically significant (Figure 3B). These results indicate that the T_3_ hormone downregulated *AFP* during hepatic progenitor differentiation into hepatocytes.

At the DE stage, we observed the dramatic upregulation of *DIO3* with the subsequent decrease in upcoming timepoints. After day 10, the expression of DIO3 decreased in both treated and nontreated cells, and at day 22, there was no difference between the conditions indicating no influence of the T_3_ hormone on *DIO3* expression (Figure 3C).

An increase in *AAT* expression in nontreated cells was detected already at day 14 compared to day 10, while in the T_3_-treated cells, it increased only by day 22. However, there was no difference in *AAT* expression by the end of the differentiation between the treated and nontreated cells (Figure 3D).

We did not observe any significant change in the total *HNF4A* expression level during the differentiation (Figure 3E). We assessed the *HNF4A* liver isoform mRNA and found its upregulation in all the conditions after the DE stage of the differentiation. It sharply increased at day 10 with a decrease at day 14 in all the conditions. Furthermore, we did not observe any change or difference in its expression by T_3_ treatment (Figure 3F).

TH responsive *THRSP* (or *SPOT14*) was significantly upregulated at day 14 in both conditions and day 22 in T_3_-treated cells. In contrast, nontreated cells did not show an increased level of *THRSP* (Figure 3G). An expected increase in the mature marker *THRSP* expression by T_3_ treatment indicates the sensitivity of the cells to the TH and an enhancement of the lipid metabolism function of HLCs [40].

#### 2.3.3. Protein Expression in GM23720B-Derived Cells

**Figure 4:**
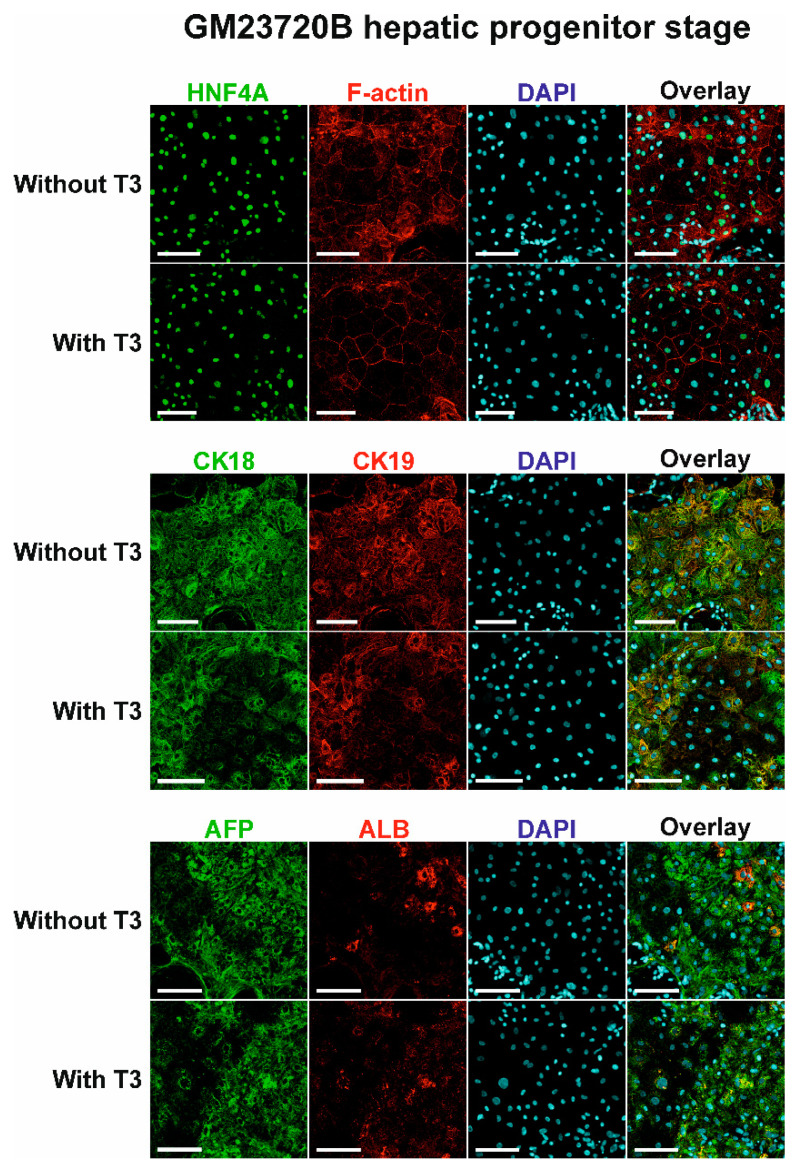
Expression of HNF4A, F-actin, CK18, CK19, AFP, and ALB proteins in GM23720B-derived cells at day 13 (hepatic progenitor stage) of the differentiation with or without T_3_ hormone in differentiation medium. Nuclei of cells were stained with DAPI (blue). Proteins of interest were stained either with Alexa Fluor 488 (HNF4A, CK18, and AFP), shown in green, or with Alexa Fluor 594 (F-actin, CK19, and ALB), shown in red. Scale bars = 100 μm.

The protein expression pattern of the GM23720B-derived hepatic cells was studied using two methods: immunohistochemical staining with further visualization using confocal microscopy and Western blotting. Staining of GM23720B-derived hepatic progenitors did not show the difference between T_3_-treated and nontreated cells, both of which had bright staining signals for HNF4A, F-actin, CK18, CK19, AFP, and ALB (Figure 4). It is worth noting that F-actin staining showed polarized polygonal cell morphology after T_3_ treatment.

**Figure 5:**
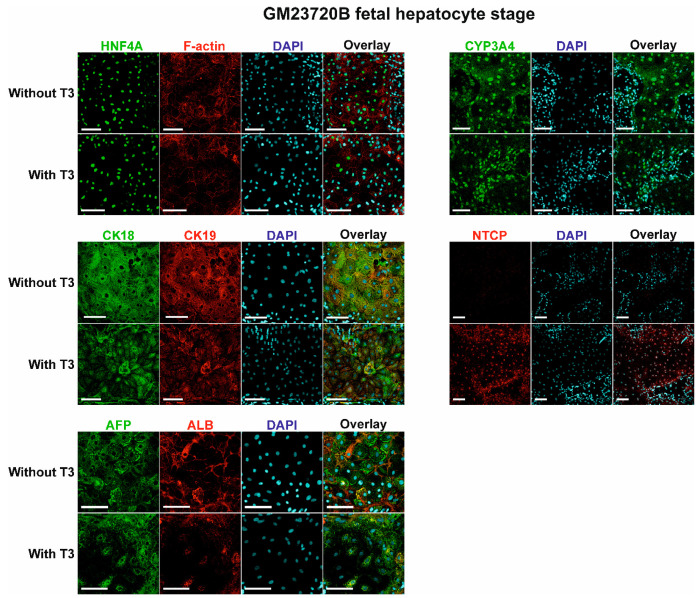
Expression of HNF4A, F-actin, CYP3A4, CK18, CK19, NTCP, AFP, and ALB proteins in GM23720B-derived cells at day 17 (fetal hepatocyte stage) of the differentiation with or without T_3_ hormone in differentiation medium. Nuclei of cells were stained with DAPI (blue). Proteins of interest were stained either with Alexa Fluor 488 (HNF4A, CYP3A4, CK18, and AFP), shown in green, or with Alexa Fluor 594 (F-actin, CK19, NTCP, and ALB), shown in red. Scale bars = 100 μm.

Staining of GM23720B-derived fetal hepatocytes showed that T_3_ treatment did not affect the expression of HNF4A, CK18, CK19, and CYP3A4 protein expression. T_3_-treated cells exhibited a polarized polygonal morphology, as seen by F-actin staining and seemed to have weaker CK19, AFP, and ALB signals than the nontreated cells (Figure 5). However, treatment with T_3_ hormone dramatically increased the positive NTCP (a sodium/bile acid cotransporter encoded by solute carrier family 10 member 1) signal (Figure 5).

**Figure 6:**
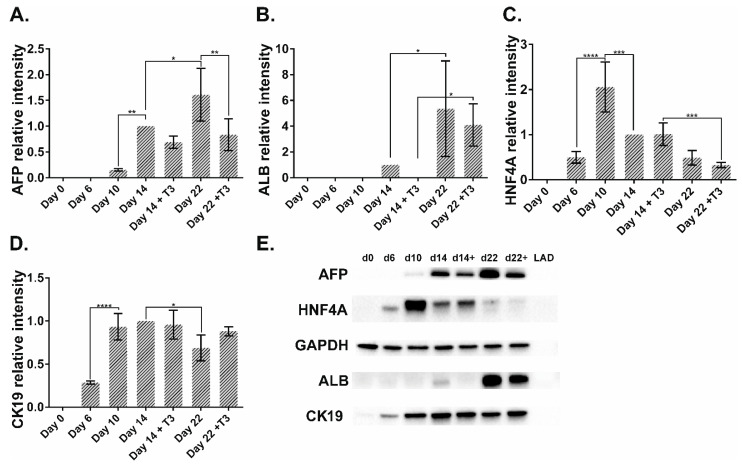
Western blotting analysis of AFP, ALB, HNF4A, and CK19 proteins in GM23720B cells and their derivatives during the hepatic differentiation at five timepoints. (**A**–**D**) The relative protein intensity normalized against GAPDH. The relative intensity at day 14 was set to 1. *n* = 3 biological repeats. Error bars are SD. One-way ANOVA followed by Sidak’s multiple comparisons test was used to compare between any pairs. Statistical significance * adjusted *p* < 0.05, ** adjusted *p* < 0.01, *** adjusted *p* < 0.001, and **** adjusted *p* < 0.0001 in comparison with SC are shown above bars. (**E**) Representative membrane picture of three independent experiments.

Western blotting analysis demonstrated that without the T_3_ treatment, AFP protein expression rose on day 14 and further increased by day 22 (Figure 6A). Cells treated with T_3_ possessed significantly lower level of AFP on day 22 than nontreated cells (Figure 6A). ALB levels in both treated and nontreated cells increased from day 14 to day 22. On day 22 there was no difference in ALB intensity between T_3_-treated and nontreated cells (Figure 6B). The relative intensity of HNF4A (all isoforms) rose at day 10 and then decreased by day 14 in the case of nontreated cells. T_3_-treated cells decreased the HNF4A intensity from day 14 to day 22 (Figure 6C). CK19 was upregulated at day 10 and then decreased by day 22 in nontreated cells. T_3_ treatment did not change CK19 protein expression (Figure 6D).

The postnatal repression of fetal-specific liver genes is one of the key mechanisms for liver maturation [41]. A recent study dedicated to the enhancement of hiPSC-derived HLCs maturity and functionality showed the improvement of metabolic functions, but the expression of *AFP* increased at the stage of hepatic progenitors and remained high until the end of the differentiation [42]. Previously, T_3_ was found to decrease AFP production and increase ALB production in mouse fetal liver cells [43]. In our protocol, the fold induction of *ALB* expression in hiPSC-derived HLCs was higher than in a previously published study using growth factor cocktail for the hepatic differentiation of hPSC [44]. Although, in our study, T_3_ did not significantly decrease the mRNA expression of *AFP* by the end of the differentiation, the treatment with T_3_ resulted in a decrease of AFP protein level in HLCs. We speculate that it might be affected by the increase of post-transcriptional AFP repression indirectly caused by T_3_ action. However, the suppressive effect of T_3_ on AFP expression is visible at the stage of fetal hepatocytes when cells were subjected to a higher concentration of T_3_. The hepatocyte-specific NTCP is an essential membrane transporter for bile acids [45]. In the current study, the upregulation of NTCP may indicate the promotion of hepatic maturation by T_3_. The induction of NTCP is highly important for the liver cell model for drug research due to its role in various liver diseases [45] and hepatitis B and D viruses entry [46]. However, T_3_ did not regulate the major serum protein ALB.

## 3. Materials and Methods

### 3.1. Cell Line

We purchased hiPSC line GM23720B from Coriell Institute (USA). Cell culture was performed on Matrigel matrix (BD Biosciences) with daily mTeSR™1 (STEMCELL™ Technologies) medium change. Subculture was done every three–four days using Versene solution 1:5000 (Invitrogen, 15040033) for cell detachment.

### 3.2. Hepatic Differentiation

Stem cells were differentiated to DE in RPMI-1640 medium (Gibco, 31870–025, Carlsbad, CA) supplemented with 1 × GlutaMAX™ (Gibco, 35050–038), 100 ng/mL activin A (AA) (PeproTech, 120-14E), and 1 × B-27 (Gibco, 17504–044) as described previously [19]. At day 5 or 6 DE cells were detached using enzyme-free cell dissociation buffer (Gibco, 13151-014) for 15 min at 37 °C and transferred on the laminin-521 (LN521, Biolamina) coating by following a previously published protocol [5]. LN521 dilution was prepared in 1 × DPBS with Ca^+^ and Mg^+^ (final concentration is 10 µg/mL) and incubated in culturing wells either overnight at +4 °C (slow coating) or for two hours at +37 °C (fast coating). DE cells were detached with the enzyme-free Cell Dissociation Buffer (Gibco, 13151-014) for 15 min at +37 °C. Then they were seeded on LN521 at the density 7.47 × 10^4^ cells/cm^2^ and cultured in Hepatocyte Culture Medium (HCM™ SingleQuots™ Kit; Lonza CC-4182, without rhEGF and gentamicin-amphotericin-1000) supplemented with 5 ng/mL fibroblast growth factor 4 (FGF4, PeproTech, 100-31), 10 ng/mL bone morphogenetic protein 2 (BMP2, Pepro-Tech, 120-02), and 10 ng/mL BMP4 (PeproTech, 120-05) for four days with daily medium change to obtain hepatic progenitors. Then hepatic progenitors were cultured in HCM supplemented with 10 ng/mL hepatocyte growth factor (HGF, PeproTech, 100-39), 10 ng/mL Oncostatin M (OSM, PeproTech, 300-10T), and 0.1 mM Dexamethasone (DEX, Sigma-Aldrich, D4902) for four days to get immature hepatocytes. Culture medium was renewed every second day. These cells were cultured in HCM supplemented with 10 ng/mL OSM, 25 ng/mL bone morphogenetic protein 7 (BMP7, PeproTech, 120-03), 0.1 mM DEX, and 10 µM DAPT (Tocris, 2634) in DMSO for four days. Culture medium was changed every second day. Finally, the cells were cultured in HCM medium supplemented with 25 ng/mL BMP7, 0.1 mM DEX, and 10 µM DAPT in DMSO for five days. We changed the culture medium every second day. To find the optimal T_3_ hormone concentration for the improvement of hepatic maturation, we first tested four concentrations of triiodothyronine (T_3_; Sigma, T6397-100MG) hormone in HBSS buffer (Gibco, 14025-050) (1 nM, 10 nM, 100 nM and 1000 nM) and three administration timing intervals (from day 9 to day 22, from day 13 to day 22, and from day 17 to day 22). After qPCR assessment of hepatic markers, we chose to use 1000 nM T_3_ from hepatic progenitor stage for four days followed by 10 nM T_3_ for the rest of differentiation period (Figure S1A). During T_3_ treatment, 1000 nM or 10 nM T_3_ in HBSS was added into cell culture wells daily.

### 3.3. RNA Isolation and cDNA Conversion

We isolated RNA from cells at five timepoints of the differentiation: stem cells (day 0), DE stage (day 6), hepatoblasts stage (day 10), fetal hepatocytes (day 14), and adult hepatocyte-like cell stage (day 22). Cells were lysed using TRI-reagent (Zymo-research, R2050-1-50). Thereafter, RNA was isolated using a Direct-zol RNA MicroPrep kit (Zymo-research, R2060) accordingly to the manufacturer’s instruction. Human fetal liver (HFH) mRNA was purchased from BioChain (lot numbers are HFH1: A601605, HFH2: A601607, and HFH3: B210099) and primary human hepatocyte (PHH) mRNA samples were isolated from PHHs (BD Gentest™, PHH1: #454503, lot 95; PHH2: #454503, lot 99; PHH3: #454426, lot 453251202). RNA concentration was measured with NanoDrop™ One (Thermo Scientific). The cDNA conversion was made with a High-Capacity cDNA reverse transcription kit (Applied Biosystems, 4368814).

### 3.4. Quantitative PCR (qPCR)

A StepOnePlus Real-Time PCR System (Applied Biosystems) machine was used for performing qPCR reactions of the cDNA samples using either a PowerUp SYBR Green Master Mix (Applied Biosystems, A25741) or TaqMan Gene Expression Master Mix (Applied Biosystems, 4369016). After qPCR using PowerUp SYBR Green Master Mix the target specificity was assessed by melting curves. For the relative gene expression calculation, a housekeeping gene ribosomal protein, large, P0 (*RPLP0*) was used. All the used primers and TaqMan^®^ Gene Expression Assay mixes are listed in Tables S1 and S2, respectively. All primers were designed by Primer Express v2.0 software (Applied Biosystems) [5], and they were synthesized by Oligomer Oy (Helsinki, Finland) or Metabion (Planegg, Germany). The relative quantification of each target gene in comparison with the housekeeping gene was made by a standard curve method based on a published mathematical model [47]. The standard curve method calculates the actual amplification efficiency, which is then taken into the calculation of relative gene expression. The mean expression values in undifferentiated stem cells (Day 0) were set as one and used as a reference for the calculation of the relative gene expressions in followed timepoints.

### 3.5. Protein Isolation

Protein samples were collected from cells at five time points: stem cells (day 0), DE stage (day 6), hepatoblast stage (day 10), fetal hepatocyte stage (day 14), and adult HLC stage (day 22). Cells were lysed using a 1x protease inhibitor cocktail (Sigma Aldrich, P8340) in Pierce RIPA buffer (Thermo Scientific, 89901) for 10 min on ice. Thereafter, samples were centrifuged at 14000 g for 15 min at +4 °C to pellet the cell debris. The supernatant was collected and frozen. Protein concentration was calculated based on absorbance measurement using a Pierce BCA Protein Assay Kit (Thermo Scientific, 23227) according to the instruction. The absorbance was detected on a Varioscan LUX device with Scanit 6.0 program.

### 3.6. Western Blot Analysis

Protein samples were diluted in 4 × Laemmli buffer (Bio-rad, 1610747) with 10% β-mercaptoethanol. Protein samples (5 µg) and PageRuler Plus Prestained Protein Ladder (Thermo Fisher Scientific, 26619, 5 µL) were loaded into the Mini-PROTEAN TGX Stain-free precast gel 4–20% (Bio-rad, 4568096) and run at voltage 125 V for approximately 90 min in a Mini-PROTEAN device. Thereafter, the gel was electroblotted to a 0.2 µm nitrocellulose membrane using a Trans-Blot Turbo Mini Nitrocellulose Transfer Pack (Bio-rad, 1704158) on a Trans-Blot Turbo Blotting System using a built-in program. Blocking was accomplished in a 5% milk solution. The membranes were incubated with the primary antibody dilution in 1% milk overnight at +4 °C and, thereafter, with the secondary antibody dilution in 1% milk for 1 h at +25 °C. After each antibody incubation stage, membranes were washed with TBS-T three times for 15 min. The antibodies used in Western blotting in the current study are listed in Table S3. A Clarity Western ECL Substrate kit (Bio-Rad, 1705061) was used for visualization of the secondary antibody signals. Images were taken on a ChemiDoc MP imager (Bio-Rad). Relative quantities of proteins were calculated using Image Lab 6.0.1 (Bio-Rad).

### 3.7. Immunofluorescent Staining

For the immunostaining, cells were cultured and differentiated either in 8-well Lab-Tek^®^ Chamber Slide™ systems (Nunc, 177445, Roskilde, Denmark) or in black 96-well μ-plates (ibid, 89626, Planegg/Martinsried, Germany). The cells were fixed at the stage of undifferentiated SCs (day 0), DE (day 5), hepatic progenitor stage (day 13), and fetal hepatocyte stage (day 17). Fixation with 4% paraformaldehyde for 10 min was followed by permeabilization either with 0.1% Triton X-100 or with 0.5% Saponin for 10 min. The blocking step was made by 10% normal goat or donkey serum (Millipore, Burlington, MA) for 1 h. Primary antibody staining was conducted for 24 h at +4 °C, and negative controls included staining with non-immunized normal rabbit IgG (Peprotech 500-P00), goat IgG (Santa Cruz Biotechnology, sc-2018), and mouse IgG (Peprotech 500-M00). Thereafter, cells were stained with the secondary antibodies conjugated with Alexa Fluor 594 or Alexa Fluor 488 (Invitrogen, 1:400) for 1 h at room temperature. Cell nuclei were stained with DAPI (Sigma-Aldrich, D8417, 12.5 μg/mL in MilliQ water) for 2 min. Cells in chamber slides were mounted with a Vectashield mounting medium (Vector, H-1500). Samples in 96-well μ-plates were filled up with 1xDPBS. The protein expression was visualized using a confocal microscope Leica TCS SP5II HCS A with aHCX PL APO 20×/0.7 objective. DAPI was excited with UV (diode 405 nm/50 mW), Alexa Fluor 488 with an Argon 488 nm laser, and Alexa Fluor 594 with a DPSS (561 nm/20 mW) laser. Primary and secondary antibodies are listed in Table S4.

### 3.8. Statistical Analysis

Statistical analyses were performed using GraphPad Prism 8 software. Statistical significance was determined by one-way analysis of variance (ANOVA) followed by Sidak’s multiple comparisons test. Differences of adjusted *p* < 0.05 (*), adjusted *p* < 0.01 (**), adjusted *p* < 0.001 (***), and adjusted *p* < 0.0001 (****) were considered significant.

## 4. Conclusions

Taken together, T_3_ at the chosen concentrations selectively influenced the expression of fetal marker AFP and maturation markers THRSP and NTCP during the differentiation of hiPSCs into HLCs in vitro. To make a thorough examination of the T_3_ action during hepatic differentiation and maturation of hiPSCs, more liver markers and functions need to be measured, such as drug metabolizing enzymes, glycogen production, and albumin secretion. Based on the current results, we cannot make a concrete conclusion that the treatment of T_3_ can improve hepatic maturation. The physiological changes during the perinatal period involve not only the surge of THs, but also changes in other hormones and growth factors. The use of T_3_ during in vitro hPSC differentiation may not be sufficient to generate mature hepatocytes. Complete hepatic maturation requires a cocktail of optimal hormones and growth factors. In addition, the T_3_ effect on the expression of genes and proteins need to be further studied. The use of TR antagonists and gene knockout cells can elucidate the role of TRs and downstream molecules. Our recent study offers a method to efficiently generate knockout hPSC lines by CRISPR/Cas 9 genome editing technology [48]. Previously, liver gene regulation by TH has been studied in human fatty liver [49] and rodents [50,51]. To the best of our knowledge, this is the first study addressing T_3_ influence on hepatic differentiation of hiPSCs in vitro.

## Data Availability

Data is contained within the article or supplementary material.

## Supplementary Materials

The followings are available online at https://www.mdpi.com/article/10.3390/ph14060544/s1, Figure S1. Hepatic differentiation of the hiPSC line GM23720B. A: Scheme of the optimized differentiation protocol. Beginning from day 10 cells were differentiated either without T_3_ hormone (a) or with T_3_ hormone (b) in the medium. B: The morphological changes of GM23720B and their derivatives during hepatic differentiation. Pictures were taken at five timepoints (day 0, day 6, day 10, day 14, and day 22). Scale bars = 100 μm. Figure S2. The preliminary assessment of the T_3_ action at different concentrations and at different timepoints. The mRNA expression patterns of the mature hepatic (*ALB*, *THRSP*, and *AAT*), fetal hepatic (*AFP*), and hepatic progenitor (*CK19*) specific markers during hepatic differentiation of GM23720B cells. Relative gene expression was measured by qPCR and normalized with the *RPLP0* housekeeping gene. Fold inductions were calculated with the reference to the undifferentiated stem cell samples (Day 0). N = 1 biological repeat. HFH: human fetal hepatocyte; PHH: primary human hepatocyte. Figure S3. The comparison of two time intervals (from day 9 to day 13 and from day 13 to day 17) of the T_3_ administration. The T_3_ action was assessed by the mRNA expression patterns of the mature hepatic (*ALB*, *CYP3A4*, and *THRSP*), fetal hepatic (*AFP*), and hepatic progenitor (*CK19*) specific markers during 17 days of the hepatic differentiation of GM23720B cells. Relative gene expression was measured by qPCR and normalized with the *RPLP0* housekeeping gene. Fold inductions were calculated with the reference to the undifferentiated stem cell samples (Day 0). N = 3 biological repeats. Error bars are SD. One-way ANOVA followed by Sidak’s multiple comparisons test was used to compare between any pairs. Statistical significance * adjusted *P* < 0.05 and **** adjusted *P* < 0.0001 in comparison with Day 0 are shown above bars. Statistically significant differences * adjusted *P* < 0.05 and **** adjusted *P* < 0.0001 between days of the differentiation are shown above lines.
