# Supplementary material for: Thyroid Hormone Effect on the Differentiation of Human Induced Pluripotent Stem Cells into Hepatocyte-Like Cells
Source: Pharmaceuticals (Basel). 2021 Jun 7;14(6):544. doi: 10.3390/ph14060544 (PMC8230271; doi:10.3390/ph14060544)
Supplement: Supplementary file 1 [file pharmaceuticals-14-00544-s001.zip › pharmaceuticals-1228259-supplementary.pdf]

Table S1. Primers used in qPCR.

Gene	Accession	Size (bp)	Sequence (5′ to 3′)
RPLP0	NM_001002.3<br>NM_053275.3	74	F: AATCTCCAGGGGCACCATT<br>R: CGCTGGCTCCCACTTTGT
AFP	NM_001134.1	79	F: GTTGCCAACTCAGTGAGGACAA<br>R: CTGATACATAAGTGTCCGATAATAATGTCA
ALB	NM_000477.3	85	F: AAGTGGGCAGCAAATGTTGTAA<br>R: AACTGGTTCAGGACCACGGATA
CK19	NM_002276.4	142	F: AGCATGAAAGCTGCCTTGGA<br>R: CCTGATTCTGCCGCTCACTATC
DIO3	NM_001362.3	94	F: GCACTTGGTTGGAACGCTAT<br>R: AGCCCACCAAGTTCAGTCAA
THRSP	NM_003251.3	61	F: GGACACTAGGGAAGATCCCTTCA<br>R: CTCCGCCGACCTCATCAAA

Table S2. TaqMan gene expression assyas used in qPCR.

Gene	TaqMan® Gene Expression assay ID
RPLP0	Hs99999902_m1
HNF4A (total)	Hs00230853_m1
HNF4A (liver)	Hs00604431_m1
CYP3A4	Hs00604506_m1

Table S3. Antibodies used in Western blotting.

**Antibody type**	**Antibody**	**Manufacturer, catalog #**	**RRID**	**Dilution ratio**
Primary	AFP	Sigma-Aldrich, A8452	AB_258392	1:5500
	ALB	Bethyl Laboratories, A80-229A	AB_67018	1:3000
	HNF4A	Santa Cruz Biotechnology, C-19, 6556	AB_2117025	1:400
	CK19	Santa Cruz Biotechnology, A53-B/A2, sc-6278	AB_627851	1:1000
	GAPDH	Cell Signaling Technology, 14C10, 2118	AB_561053	1:1000
Secondary	Rabbit anti-goat IgG HRP	Thermo Fisher Scientific, 81-1620	AB_2534006	1:5000
	Goat anti-mouse IgG HRP	Thermo Fisher Scientific, 62-6520	AB_88369	1:4000
	Goat anti-rabbit IgG HRP	Thermo Fisher Scientific, 65-6120	AB_2533967	1:5000

Table S4. Antibodies used in Immunostaining.

**Antibody**	**Manufacturer and catalog number**	**RRID**	**Dilution ratio**
Rabbit anti-OCT4	Santa Cruz Biotechnology sc-9081	AB_2167703	1:250
Goat anti-NANOG	R&D Systems AF1997	AB_ 355097	1:50
Rabbit anti-HNF4A	Sigma-Aldrich HPA004712	AB_1079075	1:50
Goat anti-SOX17	R&D Systems AF1924	AB_355060	1:25
Goat anti-HNF3B	Santa Cruz Biotechnology sc-6554	AB_2262810	1:25
Mouse anti-CXCR4	R&D Systems MAB172	AB_ 2089399	1:50
Mouse anti-Cytokeratin 18 (CK18)	Abcam ab7797	AB_306086	1:200
Rabbit anti-Cytokeratin 19 (CK19)	Abcam ab52907	AB_869877	1:250
Rabbit anti-ALB	Abcam ab2406	AB_303048	1:1000
Rabbit anti-CYP3A4	Bio-Rad AHP622Z	AB_2090332	1:600
Goat anti-NTCP	Santa Cruz Biotechnology sc-107030	AB_2188250	1:50
Mouse anti-AFP	Sigma-Aldrich A8452	AB_258392	1:500
Donkey anti-goat IgGAlexa Fluor 594	Invitrogen, A11058	AB_142540	1:200
Goat anti-rabbit IgG Alexa Fluor 488	Life Technologies, A11008	AB_143165	1:200
Goat anti-rabbit IgG Alexa Fluor 594	Invitrogen, A11012	AB_141359	1:200
Goat anti-mouse IgG Alexa Fluor 488	Termo Fisher Scientific A11001	AB_2534069	1:200

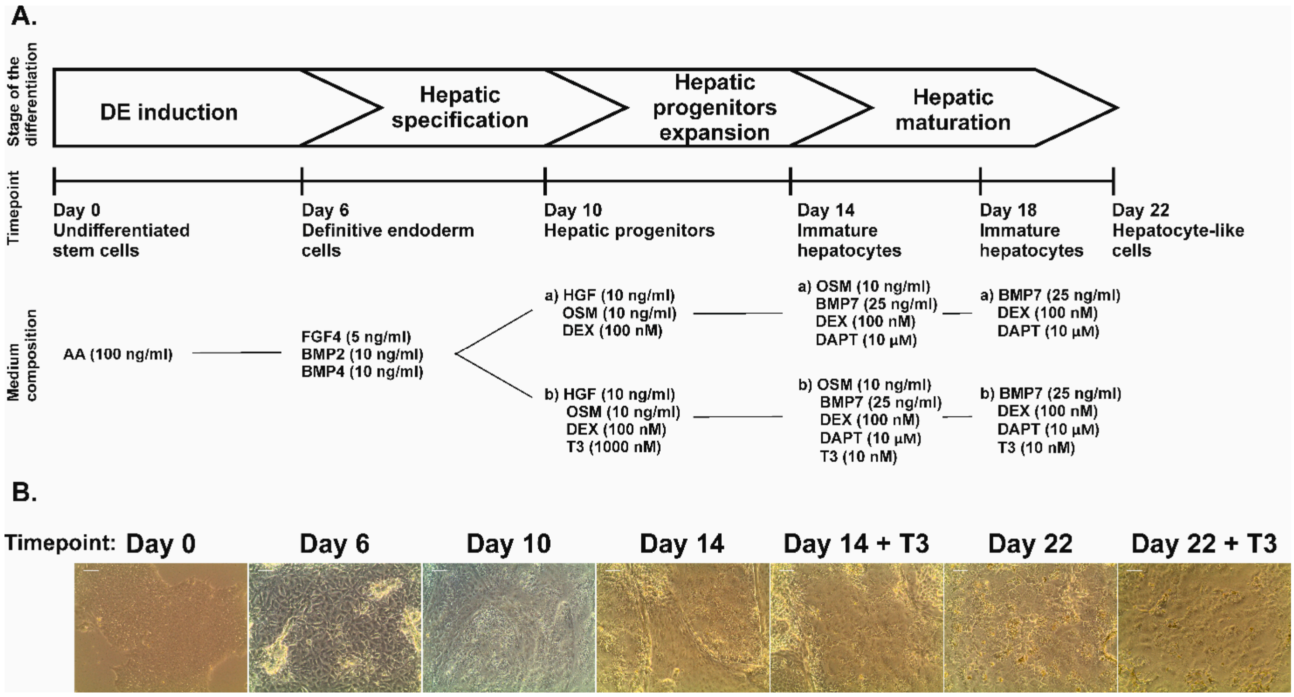

Figure S1. Hepatic differentiation of the hiPSC line GM23720B. A: Scheme of the optimized differentiation protocol. Beginning from day 10 cells were differentiated either without T_3 hormone (a) or with T_3 hormone (b) in the medium. B: The morphological changes of GM23720B and their derivatives during hepatic differentiation. Pictures were taken at five timepoints (day 0, day 6, day 10, day 14, and day 22). Scale bars = 100 µm.

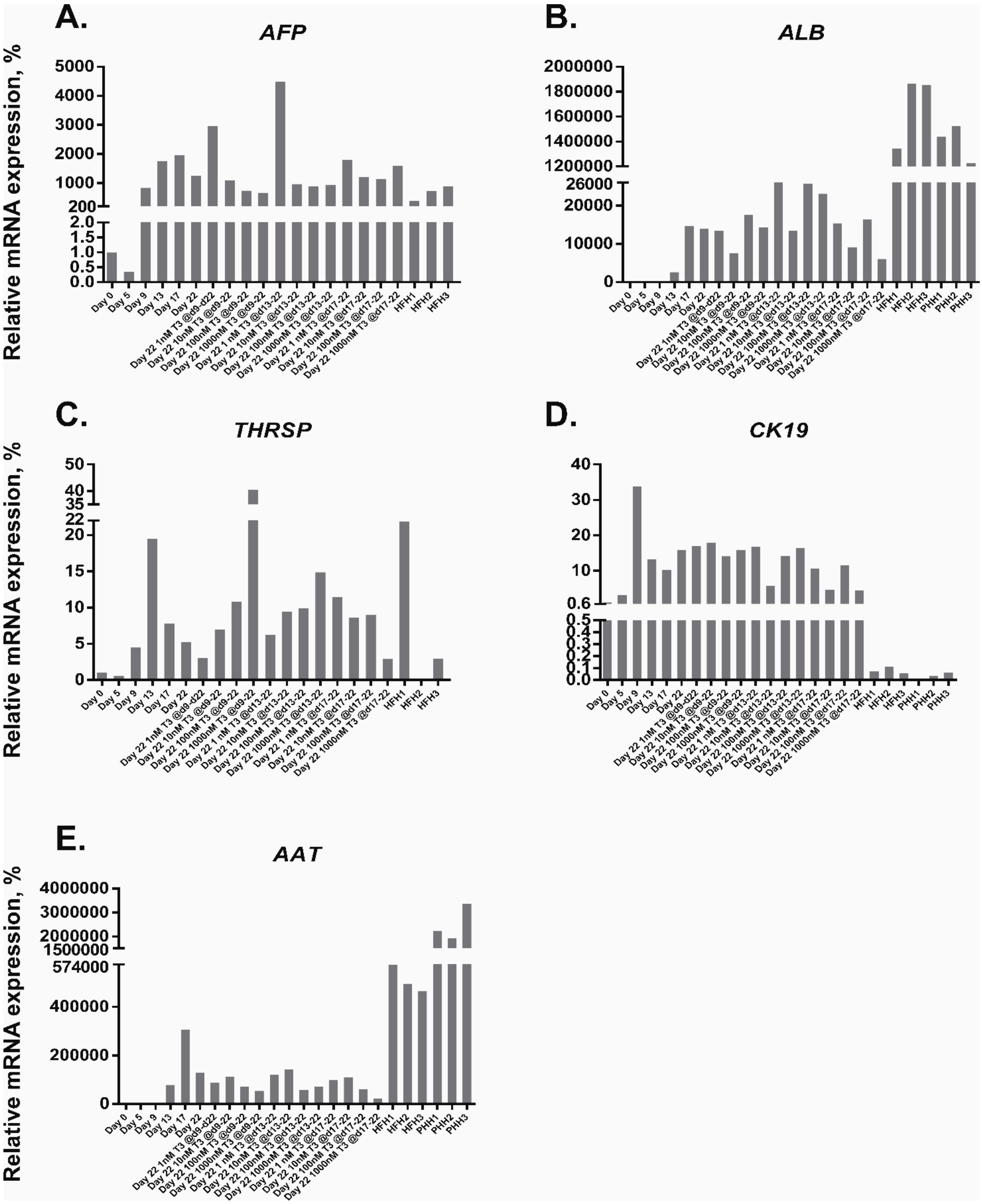

Figure S2. The preliminary assessment of the T_3 action at different concentrations and at different timepoints. The mRNA expression patterns of the mature hepatic (*ALB*, *THRSP*, and *AAT*), fetal hepatic (*AFP*), and hepatic progenitor (*CK19*) specific markers during hepatic differentiation of GM23720B cells. Relative gene expression was measured by qPCR and normalized with the *RPLP0*

housekeeping gene. Fold inductions were calculated with the reference to the undifferentiated stem cell samples (Day 0). N = 1 biological repeat. HFH: human fetal hepatocyte; PHH: primary human hepatocyte.

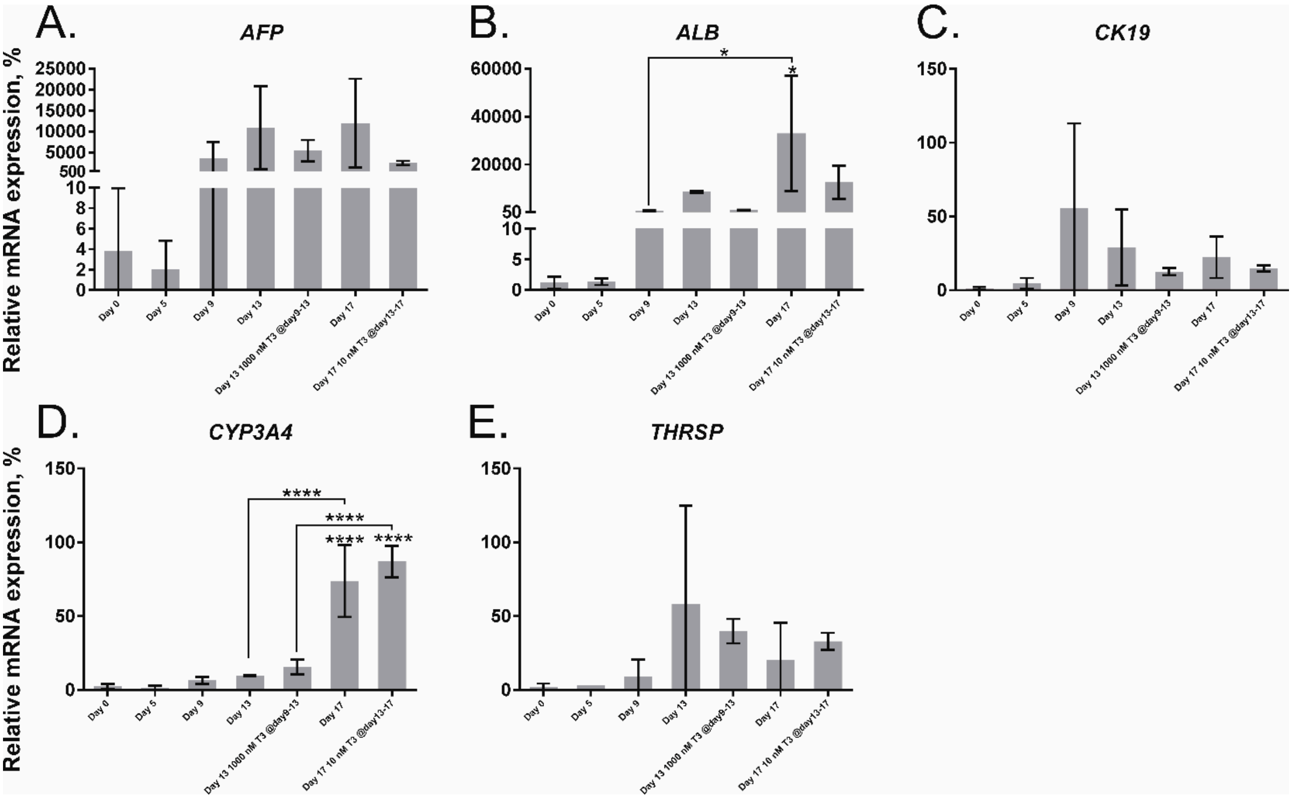

Figure S3. The comparison of two time intervals (from day 9 to day 13 and from day 13 to day 17) of the T_3 administration. The T_3 action was assessed by the mRNA expression patterns of the mature hepatic (*ALB*, *CYP3A4*, and *THRSP*), fetal hepatic (*AFP*), and hepatic progenitor (*CK19*) specific markers during 17 days of the hepatic differentiation of GM23720B cells. Relative gene expression was measured by qPCR and normalized with the *RPLP0* housekeeping gene. Fold inductions were calculated with the reference to the undifferentiated stem cell samples (Day 0). N = 3 biological repeats. Error bars are SD. One-way ANOVA followed by Sidak's multiple comparisons test was used to compare between any pairs. Statistical significance * adjusted $P < 0.05$ and **** adjusted $P < 0.0001$ in comparison with Day 0 are shown above bars. Statistically significant differences * adjusted $P < 0.05$ and **** adjusted $P < 0.0001$ between days of the differentiation are shown above lines.
